# Hippocampus segmentation after brain tumor resection via postoperative region synthesis

**DOI:** 10.1186/s12880-023-01087-2

**Published:** 2023-09-28

**Authors:** Changjuan Tao, Difei Gu, Rui Huang, Ling Zhou, Zhiqiang Hu, Yuanyuan Chen, Xiaofan Zhang, Hongsheng Li

**Affiliations:** 1 Department of Radiation Oncology, Zhejiang Cancer Hospital, Hangzhou Institute of Medicine (HIM), Chinese Academy of Sciences,, Hangzhou, China; 2 Interactive Intelligence (CPII) Limited, Hong Kong SAR, China; 3 SenseTime Research, Shanghai, China; 4 Department of Radiation oncology, Dongguan People’s Hospital, Dongguan, China; 5 Qing Yuan Research Institute, Shanghai Jiao Tong University, Shanghai, China; 6 Department of Electronic Engineering, The Chinese University of Hong Kong, Hong Kong SAR, China

**Keywords:** Automatic hippocampus segmentation, Postoperative image synthesis, Variational generative adversarial network, Radiotherapy

## Abstract

**Purpose:**

Accurately segmenting the hippocampus is an essential step in brain tumor radiotherapy planning. Some patients undergo brain tumor resection beforehand, which can significantly alter the postoperative regions’ appearances and intensity of the 3D MR images. However, there are limited tumor resection patient images for deep neural networks to be effective.

**Methods:**

We propose a novel automatic hippocampus segmentation framework via postoperative image synthesis. The variational generative adversarial network consists of intensity alignment and a weight-map-guided feature fusion module, which transfers the postoperative regions to the preoperative images. In addition, to further boost the performance of hippocampus segmentation, We design a joint training strategy to optimize the image synthesis network and the segmentation task simultaneously.

**Results:**

Comprehensive experiments demonstrate that our proposed method on the dataset with 48 nasopharyngeal carcinoma patients and 67 brain tumor patients observes consistent improvements over state-of-the-art methods.

**Conclusion:**

The proposed postoperative image synthesis method act as a novel and powerful scheme to generate additional training data. Compared with existing deep learning methods, it achieves better accuracy for hippocampus segmentation of brain tumor patients who have undergone brain tumor resection. It can be used as an automatic contouring tool for hippocampus delineation in hippocampus-sparing radiotherapy.

## Introduction

**Fig. 1:**
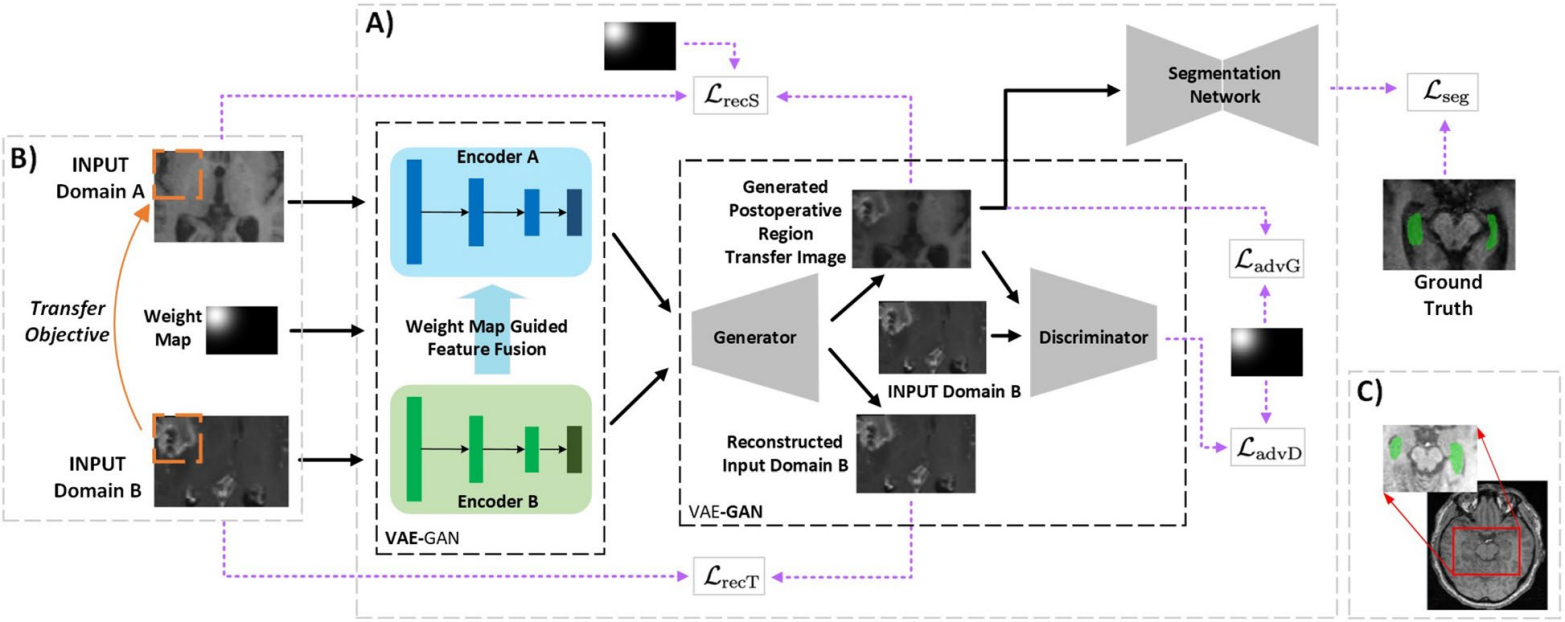
**A** Our proposed brain tumor image generation architecture (VAE-GAN) with joint image segmentation. It consists of two components VAE and GAN in which encoder-decoder architectures are utilized. Detailed schematics are explored in the methods section. **B** Nasopharyngeal carcinoma brain MR images (top) and brain tumor MR images (bottom). The fake image is generated by transferring the postoperative region (indicated by the orange dashed bounding box) from the bottom image to the top image. **C** MR brain cross-section. The hippocampus within the enlarged cropped region is labeled with green markers

Radiotherapy is an effective treatment for patients with brain tumors. During the planning for radiotherapy, a series of normal organs (organs-at-risk) need to be spared from radiation, especially the hippocampus. Hippocampus, highlighted in Fig. 1C is a small S-shaped structure within the temporal lobe that can be identified as a layer of densely packed neurons [1], which plays an important role in the formation of new memories. If the radiation with too high dose injures the hippocampus, it would influence a person’s learning and memory functionalities as well as their ability to remember directions, locations and orientations [2]. Accurately delineating the hippocampus is an essential step in radiotherapy planning. According to report RTOG 0933, hippocampus-sparing radiotherapy can provide better preservation of memory and cognitive function [3].

The treatment manner for patients with brain tumors might involve both tumor resection and radiotherapy. Due to the surgical treatment, the brain tumor images may contain postoperative regions that exhibit significant regional appearance variations as well as large intensity variations between patients, as shown in Fig. 1B. When applying state-of-the-art methods on such brain tumor MR images, their segmentation performances of the hippocampus deteriorate dramatically, which hinders their clinical applications. Therefore, accurately segmenting the hippocampus for brain tumor patients who have undergone tumor resection surgery with limited training data is still a challenging problem.

Even though more deep learning-based models have been proposed for automatic hippocampus segmentation in the last few years, these deep learning models are still heavily dependent on the training data. State-of-the-art methods mostly train models on healthy or preoperative patients from public datasets [4]. Our experiments show that such models trained in previous methods are incapable of maintaining good performance on brain tumor patients who have undergone tumor resection. Collecting more of these postoperative patients is a difficult task because manual segment hippocampus is time-consuming and prone to intra- and inter-rater variations, also, there are limited patients with a brain tumor. Recently, there has been open-source dataset such as the BraTS [5–8], this dataset contains a respectable number of brain tumor MRI scans, and involving state-of-the-art methods [9–12] for locating brain tumors. Even though these datasets have data in large amounts, they are unable to provide resected brain tumor MRI scans and the involved state-of-the-art methods do not address the postoperative region. This proposed difficult problem motivates our work by looking into generative adversarial methods.

The study aims to develop an automatic method for hippocampus segmentation on MR images for brain tumor patients who have undergone tumor resection with limited training data. We propose a postoperative image synthesis framework, illustrated in Fig. 1A, to alleviate the problem mentioned above. And we find that training the generator and segmentation network together is crucial in achieving high performance. Specifically, given a brain tumor image with postoperative regions as a reference, the generator sub-network in the proposed variational generative adversarial network (VAE-GAN) synthesizes images of brain tumor resection by transferring the postoperative region from the reference image to a regular image without surgery. The process involves utilizing coupled encoders and a mask to combine important features from the two images: task-specific post-operation region information from the reference image and valuable background information from the regular image without surgery. The generator, on the other hand, learns this information and reconstructs new realistic synthetic images. In addition, in order to make the generated images realistic, the generator would also align the intensity between the generated image and the reference image. A modified 3D SEResUNet is proposed to segment the hippocampus, and the synthetic postoperative images are used to refine our segmentation network. Overall, VAE-GAN and the segmentation network are jointly optimized during the training process. We demonstrate that our proposed end-to-end joint generation-segmentation framework can improve the segmentation performance of the hippocampus significantly on brain tumor patients. The contributions of this study can be summarized as follows:

1. We propose a novel end-to-end joint generation-segmentation framework dedicated for hippocampus segmentation of head-and-neck MR images with brain tumor resection.
2. A novel VAE-GAN is developed to synthesize MR images with postoperative region. To make the generated images more realistic, an intensity alignment module is developed to align the intensity between the synthesized image and the reference image.
3. A joint training strategy has been used to simultaneously optimize the VAE-GAN and the segmentation network so that these two tasks could promote each other.

## Background

### Hippocampus segmentation

Hippocampus segmentation is a crucial pre-radiotherapy procedure. Previously conducted manually, which is inefficient and prone to error, motivates fully automatic segmentation.

The earliest method that came close to automatic segmentation was proposed with the conventional image processing technique. One of the earliest works [13, 14] investigates the method of deformable contours and applies it to the hippocampus segmentation problem. Note that these methods are not fully automated since human-computer interactions were still required. Thereafter, atlas-based registration technique was introduced [15]. This involves the need for an atlas patch-based method in combination with labels to predict the segmentation. But these methods are computationally heavy and highly dependent on the choice of an atlas. Alternative methods such as Sub-Fields segmentation techniques [16, 17] look at the hippocampus not as a homogeneous structure but rather utilizing the ultra high-field MRI scanner to find certain bio-markers for segmentation. Although refined results, these methods do not unify on segmentation protocols. Given the well-received popularity in convolutional neural networks, deep learning-based methods have been proposed [18], in which 3D U-Net was employed as it is widely used in medical image segmentation tasks [19].

However, if the patient has experienced brain surgery, such as brain tumor resection, the latest solutions fall short on segmentation accuracy. It is challenging to obtain a large amount of MR images of this type. Thus we surveyed a collection of papers that address this problem by proposing a synthetic image generation method using a Generative Adversarial Network.

### Synthetic image generation

Generative Adversarial Network (GAN) [20] is a category of models that generate synthetic data which contains the same statistics as the given training set. GAN is popular in medical tasks playing the role of data augmentation since it unravels the frequently occurring problem of insufficient data.

CycleGAN [21] is an image generation method that is unsupervised such that data and its corresponding label no longer have to coexist. CycleGAN imposes cycle consistency, in which the forward and the backward mapping functions are inverses of each other. MUNIT [22] is an unsupervised image translation model that learns image domain styles such that images from an arbitrary domain can be transferred to this domain by using its style encoder. DiscoGAN [23] is another unsupervised GAN that implements two image domain-transfer generators. DiscoGAN uses two reconstruction losses one for each direction of domain generation and forces a one-to-one domain translation.

We aim to design a VAE-GAN in conjunction with feature maps to perform region-wise transfer and create new synthetic region transferred images that strengthen our hippocampus segmentation performance. We will show it in greater details in the section “Postoperative image synthesis”.

## Methods

### Data collection

A total number of 127 patients with nasopharyngeal carcinoma and brain tumor receiving radiotherapy from February 2009 to December 2019 in the Cancer Hospital of University of the Chinese Academy of Sciences (Zhejiang Cancer Hospital) are included in this study. T1 weighted MR images (T1WI) were acquired via Siemens MRI Machine for all patients. 12 additional T1 C+ brain tumor MR images from 2023 acquired from the same source included to measure the model’s ability on images with both test-of-time and domain differences.

Specifically, there are 48 patients with nasopharyngeal carcinoma (NC) who have not experienced tumor resection and 67 + 12 patients whose brain tumors are resected (BTR) before radiotherapy.

For the NC dataset, the in-plane resolution of the images ranged from 0.36 to 0.94 mm, with a mean value of 0.8 mm and a median value of 0.94 mm. The slice thickness ranged from 0.9 mm to 3.25 mm, with a mean value of 1.31 mm and a median value of 1 mm. For the BTR dataset, the in-plane resolution of the images ranged from 0.3 to 1.02 mm, with a mean value of 0.78 mm and a median value of 0.94 mm. The slice thickness ranged from 1 mm to 7.8 mm, with a mean value of 2.05 mm and a median value of 1 mm.

Experienced doctors manually delineate the hippocampus on the RayStation Treatment Planning System, and the annotations are reviewed by another experienced doctors to confirm the correctness of annotation.

**Fig. 2:**
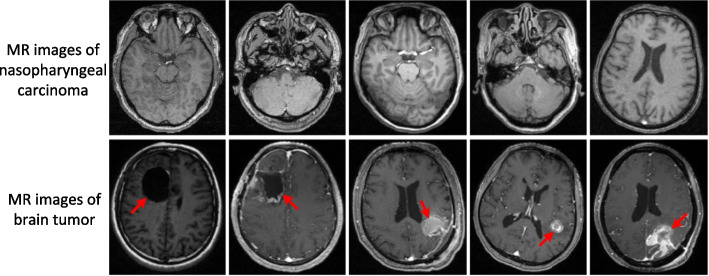
MR images of nasopharyngeal carcinoma and brain tumor. The first row is the images with nasopharyngeal carcinoma. The second row is the images with brain tumor. Usually there are some postoperative regions on brain tumor images that exhibit significant appearance variations and intensity variations

### Problem formulation

This study focuses on automatic hippocampus segmentation on MR images for patients with brain tumors who have undergone tumor resection during radiotherapy. Training data from this type of patient are very difficult to obtain. Most existing methods train their models on images of healthy or preoperative patients, such as nasopharyngeal carcinoma. However, suppose we apply these methods directly to images after tumor resection surgery, the segmentation performance of the hippocampus drops dramatically because the resection surgery altered the postoperative regions’ appearances and intensity significantly (shown in Fig. 2).

**Fig. 3:**
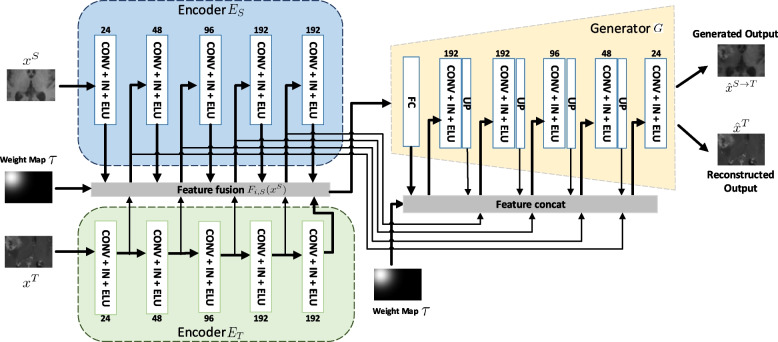
The overall pipeline of the proposed VAE-GAN framework. CONV denotes convolution layer, IN denotes instance normalization, ELU denotes exponential linear unit, FC denotes fully connected layer, and UP denotes up-sampling operation. The Schematic is reduced from 3D to 2D for visual clarity. The generated and reconstructed output depends on the intensity alignment, which is delivered by switching to the different set of parameters of the Adaptive Instance Normalization. Feature fusion is not used in the case of reconstructed output

We develop a novel joint generation-segmentation framework focusing on hippocampus segmentation for images after brain tumor resection surgery to overcome this challenge. Figure 3 is the overview of the proposed framework, which contains two main components: VAE-GAN for postoperative image synthesis and the segmentation network for hippocampus segmentation. The two components could be jointly optimized in an end-to-end manner during the training process.

### Postoperative image synthesis

The very straightforward way to deal with the lack of data is to generate more. Therefore, VAE-GAN is designed to generate the MR image after tumor resection surgery.

Specifically, a source dataset of MR images of nasopharyngeal carcinoma patients without any surgery, $\mathbb {D}_{S}=\{(x_i^S,$ $y_i^S)|i = 1, \dots , N_S\}$, and a target dataset of MR images of brain tumor patients with tumor resection, $\mathbb {D}_{T}=\{(x_i^T,$ $y_i^T)|i = 1, \dots , N_T\}$, $x_i^S$, and $y_i^S$ denote the *i*-th training images of the source domain and its corresponding segmentation mask of the hippocampus, and $N_S$ is the number of training images. $x_i^T$, and $y_i^T$ denote the *i*-th training images of the target domain and its corresponding segmentation mask for the hippocampus, and $N_T$ is the number of training images. VAE-GAN synthesizes postoperative images as additional training data by transferring the postoperative region in $\mathbb {D}_{T}$ to $\mathbb {D}_{S}$, as well as aligning the intensities between $\mathbb {D}_{S}$ and $\mathbb {D}_{T}$.

Specifically, our VAE-GAN, as illustrated in Fig. 3, is based on variational autoencoder (VAE) [24–26] and generative adversarial networks (GANs) [27, 28]. The motivation of our model comes from the fact a generator can be constructed using an encoder-decoder architecture, which learns a marginal distribution of the source and target images. Such a structure also enables any region of transfer by utilizing a mask and applying it within the encoder. The model consists of 4 sub-networks: two domain-specific image encoders $E_S$ and $E_T$, one image generator *G*, and one adversarial discriminator *D*.

**Encoder-generator.** The encoder-generator pair $\{E_T, G\}$ constitutes a VAE for the target domain, named VAE$_T$. For an input image $x^T\in \mathbb {D}_{T}$, the VAE$_T$ first maps the input image $x^T$ to a randomized latent code in a latent space $Z_T$ via encoder $E_T$ and then decodes the mapped code to reconstruct the input image via the generator *G*. We assume the codes in the latent space $Z_T$ are normally distributed. The encoder has two branches and outputs a mean vector $E^{\mu }_T(x^T)$ and a variance vector $E^{\sigma }_{T}(x^T)$, and the distribution of the latent code $z^T$ is generated by sampling a normal distribution as $q_Y(z^T|x^T) \equiv \mathcal {N}(z^T|E_{\mu , T}(x^T), E_{\sigma , T}(x^T))$. The generator takes the latent code as input and reconstructs the input image, denoted as $\hat{x}^T=G(z^T \sim q_T(z^T|x^T))$. Since images in the target domain $\mathbb {D}_{T}$ contain postoperative regions, the encoder $E_T$ also encodes appearance information around the postoperative regions and the part of the task for generator *G* is to reconstruct it.

As our goal is to synthesize postoperative images with such postoperative regions, we introduce a pre-annotated voxel-wise spatial weight map $\mathcal {T}$ to encode the location of the postoperative regions and feed it into the generator *G* to make the network aware of the surgical location. Generally, the voxels close to the postoperative region have higher weights in the weight map and vice versa. We make the weight map follows a Gaussian-like distribution. Let $(\mu _x, \mu _y, \mu _z)$ denote the centroid of the postoperative region. We use $\mathcal {T}_i$ to represent the weight of voxel *i* with the coordinate $(v_{ix}, v_{iy}, v_{iz})$ in $\mathcal {T}$:

1 $$\begin{aligned} \mathcal {T}_i = \textrm{exp}\left( -\frac{(v_{ix}-\mu _x)^2+(v_{iy}-\mu _y)^2+(v_{iz}-\mu _z)^2}{2\sigma ^2}\right) , \end{aligned}$$

where $\sigma$ is a parameter that controls the sharpness of the postoperative spatial weight map. The spatial weight map is injected into the generator via feature concatenation. An example of $\mathcal {T}$ is shown in Fig. 3.

Similarly, $\{E_S, G\}$ constitutes a VAE for the source domain, named VAE$_S$. Different from the VAE$_T$, it takes an image $x^S \in \mathbb {D}_{S}$, postoperative image features of a target domain image from the encoder $E_T$ as inputs. In addition, to make the encoder $E_S$ understand the surgical location, we also introduce the voxel-wise spatial weight map $\mathcal {T}$ to encode the location of the postoperative regions.

Generally, $E_S$ first maps $x^S$ to a latent code in a latent space $Z_S$. In order to transfer the postoperative region from the target domain to the source domain, we propose to integrate features around the postoperative region from the encoder $E_T$ into the feature maps of the encoder $E_S$ via the spatial weight map $\mathcal {T}$:

2 $$\begin{aligned} F_{i, S}(x^S) = \mathcal {T} \odot F_{i, T}(x^T) + (1 - \mathcal {T}) \odot {F}_{i, S}(x^S), \text { for } i = 1,\dots , N_e, \end{aligned}$$

where $\odot$ denotes spatial-wise multiplication, and $N_e$ is the number of the layers in the encoder, $\hat{F}_{i, S}(x^S)$ denote the *i*-th layer’s feature of the encoder $E_S$ before feature fusion, and $F_{i, T}(x^T)$ denote the *i*-th layer’s feature of the encoder $E_T$. Then the *i*-th layer’s feature after fusion contains the information from both encoders $E_S$ and $E_T$.

To transfer the postoperative region from the target domain to the source domain, we adopt a weight-sharing strategy on the generator *G* to relate the two VAEs between two domains, i.e., *G* is the shared-weights generator. For an image $x^T$ in the target domain, the generator aims to reconstruct the same image with tumor resection. Therefore, we use the L1 loss to supervise the reconstruction task as

3 $$\begin{aligned} \mathcal {L}_{\textrm{recT}} = {\Vert G(z^T) - x^T \Vert }_1. \end{aligned}$$

For an image $x^S$ in the source domain, we aim to synthesize a postoperative image by transferring the postoperative region of the image $x^T$ to the corresponding location of $x^S$ while keeping the image contents outside the postoperative region unchanged.

We first obtain a binary mask $\mathcal {M}$ of the postoperative region by thresholding the normally-distributed weight map $\mathcal {T}$ obtained above. We supervise the reconstruction of the voxels outside the postoperative region using the binary mask $\mathcal {M}$ with the L1 loss as

4 $$\begin{aligned} \mathcal {L}_{\textrm{recS}} = (1 - \mathcal {M}) \odot {\Vert G(z^S) - x^S \Vert }_1, \end{aligned}$$

where $\odot$ denotes spatial-wise multiplication.

**Intensity alignment.** The intensities between the two domains also exhibit large variations (see Fig. 5). If we share all the parameters of the generator *G*, the foreground (postoperative region) and background of the synthetic images would be inharmonious.

To align the intensities of the images, we could follow the idea of Adaptive Instance Normalization [29], where instance normalization conducts style normalization by normalizing feature statistics, i.e., the channel-wise mean and variance. [29] shows that the feature statistics can control the style of the generated image.

Therefore, we can normalize their feature statistics and consequently normalize the output image intensity by using different sets of affine parameters in instance normalization of the two sets of images. We integrate the generator *G* with instance normalization layers and two sets of different affine parameters $(\gamma ^S, \beta ^S)$ and $(\gamma ^T, \beta ^T)$, one for the source domain and the other one for the target domain.

At the training stage, each domain use their affine parameters to encode their own style (intensity) information,

5 $$\begin{aligned} \hat{x}^T= & {} G(z^T, \gamma ^T, \beta ^T), \end{aligned}$$

6 $$\begin{aligned} \hat{x}^{S}= & {} G(z^S, \gamma ^S, \beta ^S), \end{aligned}$$

where $z^S$ and $z^T$ are the latent codes from two encoders. $(\gamma ^S, \beta ^S)$ and $(\gamma ^T, \beta ^T)$ are the two sets of affine parameters in all instance normalization layers in generator *G*.

At the inference stage, we use the affine parameters $(\gamma ^T, \beta ^T)$ for images from the source domain in order to align the intensity between the two domains and make the synthetic images more harmonious.

7 $$\begin{aligned} \hat{x}^{S\rightarrow T} = G(z^S, \gamma ^T, \beta ^T). \end{aligned}$$

**Discriminator.** To properly supervise the generator *G* to synthesize realistic images, we adopt an adversarial discriminator *D* to distinguish whether an image is real ($x^T$ from the target domain with the postoperative region) or fake ($\hat{x}^{S \rightarrow T}$ transferred from the source domain). The *G* is trained to fool the adversarial discriminator *D*.

For traditional discriminators in image synthesis tasks, only an image is fed into the discriminator as input. However, the synthesized postoperative region only appears in a small image area. There is no guarantee that the postoperative region could be transferred from the target domain to the source domain in the corresponding position. We, therefore, encourage the discriminator not only to find whether the synthesized image is real or fake, but also whether the postoperative location of the synthetic image matches that of the image $x^T$ with tumor resection.

Inspired by the semantic embedding discriminator [30], which proposed a patch-based semantics embedding discriminator to tell not only real or fake but also whether the patches match their corresponding semantic labels. We design our discriminator *D* with a similar idea, where the postoperative region weight map $\mathcal {T}$ is employed to force the discriminator to focus on the postoperative region. Our discriminator takes the real images from the target domain $x^T$ or the generated images $\hat{x}^{S \rightarrow T}$ as inputs.

The discriminator creates a set of feature pyramids of different spatial scales. The feature vector at each spatial location represents a patch corresponding to the input image. *D* tries to classify whether each patch is real or fake by the predicted score for each spatial location in the feature pyramids. Specifically, for each spatial scale, we first downsample the weight map to the same spatial resolution and convert the weight map to a vector with a $1\times 1$ convolution at each spatial location, whose dimension is equal to that of the feature maps from the pyramid. We then calculate the inner product between feature vectors of feature pyramids and location embedding map to generate a postoperative region-aware matching score map. Finally, the postoperative region-aware matching score map can represent the discriminator’s confidence in the patches’ realness and drive the generator *G* to synthesize a real and spatially aligned postoperative region with the image from the target domain.

Following the previous work [30], we adopt the hinge loss as the adversarial loss,

8 $$\begin{aligned} \mathcal {L}_{\textrm{advD}}= & {} -\min (0, -1 + D(x^T, \mathcal {T}))\nonumber \\{} & {} -\min (0, -1 - D(\hat{x}^{S \rightarrow T}, \mathcal {T})),\end{aligned}$$

9 $$\begin{aligned} \mathcal {L}_{\textrm{advG}}= & {} - D(\hat{x}^{S \rightarrow T}, \mathcal {T}), \end{aligned}$$

where $x^T$ is the image from the target domain, $\hat{x}^{S \rightarrow T}$ is the image transferred from the source domain to the target domain with postoperative region, and $\mathcal {T}$ is the Gaussian-like weight map.

### Hippocampus segmentation

Since the final goal is to segment the hippocampus in MR images after brain tumor surgery, our segmentation network *S* inputs are the synthesized postoperative images and the real images from the target domain (with postoperative regions).

Unlike existing methods that treat the segmentation network and the generative adversarial networks as two separate components, we argue that the two networks could be jointly optimized to achieve optimal performance. Specifically, our VAE-GAN for synthesizing postoperative images is jointly trained with the segmentation network in an end-to-end manner. Hence, the generated images not only contain postoperative regions but also benefits the segmentation task. Moreover, the generator can act as a strong data augmentation scheme that can provide abundant training samples to improve the segmentation performance.

**Fig. 4:**
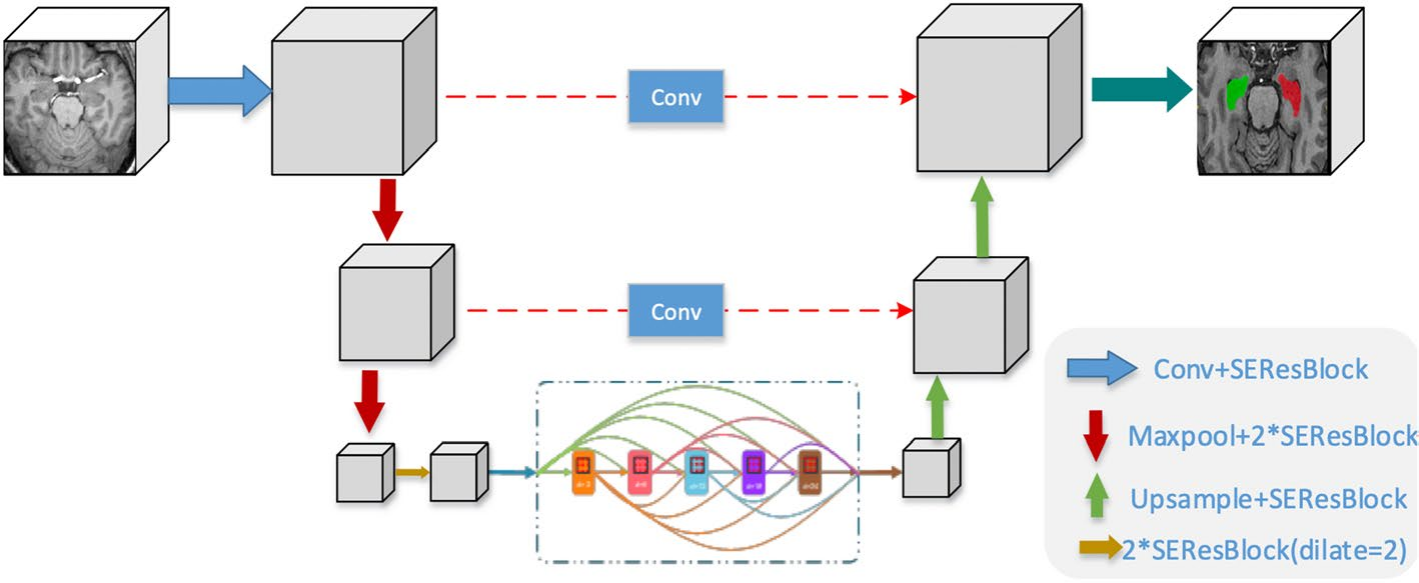
The network architecture of our proposed segmentation network

**Segmentation network.** U-Net [31] is the predominant approach in medical image segmentation because of its powerful feature learning capability. Our segmentation network is based on the 3D U-Net, which could capture the volumetric contextual information. And we further modify it for the hippocampus segmentation task.

We replace the standard Conv-BN-ReLU blocks with the squeeze-and-excitation res-blocks (SEResBlock) [32], The SEResBlock introduces channel-wise attention by adaptively re-weighting channel-wise feature responses to explicitly model the importance of each channel.

Since the hippocampus is a small region in MR images and too much downsampling operation would lead to the loss of spatial information, which hurts the segmentation performance for the hippocampus. Therefore, we only perform the downsampling twice and the upsampling twice via convolution and deconvolution with the stride of 2. However, fewer downsampling layers may lead to smaller receptive fields. In order to solve this problem, DenseASPP module [33] is inserted between the downsampling and upsampling stage. By concatenating a set of atrous convolution with different dilation rates in a dense way, DenseASPP effectively generates densely spatial-sampled and scale-sampled features while enlarging the valid receptive field. The detail of our modified 3D U-Net is shown in Fig. 4.

**Segmentation loss.** The dice loss can be formulated as

10 $$\begin{aligned} \mathcal {L}_\text {dice} = \sum \limits _{t=0}^{C}\left( 1-2 \frac{\sum y_t p_t + \epsilon }{\sum y_t +\sum p_t+\epsilon }\right) , \end{aligned}$$

where $y_t$ and $p_t$ represent the ground-truth label and model’s predictions for class *t*, respectively, and $\epsilon$ is a small value to ensure numerical stability.

The hippocampus is a small organ, leading to extreme foreground-background class imbalance. To alleviate the imbalance problem, we adopt the weighted focal loss [34] and dice loss [35] as the segmentation losses. The weighted focal loss is formulated as

11 $$\begin{aligned} \mathcal {L}_\text {focal}=-\alpha _t(1-p_t)^\gamma \text {log}(p_t), \end{aligned}$$

where $p_t$ is the model’s estimated probability that a sample is correctly classified, and $\alpha _t$ is used to balance the foreground and background. In general, the focal loss down-weights the well-classified examples and makes the training focus more on the hard examples. The hyper-parameters of the focal loss $\alpha$ and $\gamma$ are empirically set as 0.25 and 2.

The overall segmentation loss is thus defined as

12 $$\begin{aligned} \mathcal {L}_{\textrm{seg}}= \mathcal {L}_{\textrm{focal}} + \lambda \mathcal {L}_{\textrm{dice}}, \end{aligned}$$

where $\lambda$ is the loss weight to balance the two losses.

### Joint training strategy and overall losses

The segmentation network *S* (pretrained with $\mathbb {D}_{S}$ and $\mathbb {D}_{T}$), generator *G*, and discriminator *D* are trained alternatively. The generator *G* is optimized by minimizing $\mathcal {L}_{G}$ with *D* and *S* fixed,

13 $$\begin{aligned} \mathcal {L}_{G}= \lambda _{\textrm{rec}}(\mathcal {L}_{\textrm{recS}} + \mathcal {L}_{\textrm{recT}}) + \lambda _{\textrm{advG}}\mathcal {L}_{\textrm{advG}} + \lambda _{\textrm{segG}}\mathcal {L}_{\textrm{seg}}. \end{aligned}$$

The discriminator *D* is then optimized by fixing *G* and *S*, and it minimizes the loss $\mathcal {L}_{D}$,

14 $$\begin{aligned} \mathcal {L}_{D}= \lambda _{\textrm{advD}}\mathcal {L}_{\textrm{advD}}. \end{aligned}$$

The segmentation network *S* which minimizes the loss $\mathcal {L}_{S}$, as mentioned above, could be jointly optimized with *G* and *D* for better performance. Note that during training, the loss $\mathcal {L}_{S}$ is not minimized until further into the epochs, see section “Implementation details” for detailed training procedures.

15 $$\begin{aligned} \mathcal {L}_{S}= \lambda _{\textrm{segS}}\mathcal {L}_{\textrm{seg}}, \end{aligned}$$

where $\lambda _{rec}, \lambda _{advG}, \lambda _{advD}, \lambda _{segG}, \lambda _{segS}$ balances the importance of the losses.

Finally, the overall loss can be formulated as,

16 $$\begin{aligned} \mathcal {L}_{\textrm{overall}}= \mathcal {L}_{G} + \mathcal {L}_{D} + \mathcal {L}_{S}, \end{aligned}$$

## Results

### Experimental settings

To evaluate the effectiveness of our proposed hippocampus segmentation framework, we retrospectively collected 48 MR images of nasopharyngeal carcinoma patients without tumor resection and 67 MR images of brain tumor patients with tumor resection. Images from the BTR dataset are randomly divided into training and test sets with a ratio of 4:1.

We set up a four stages ablation study and observe performance changes. In addition, we also compare our method with the most representative unsupervised image-to-image translation methods: MUNIT [22], DiscoGAN [23] and CycleGAN [21].

### Implementation details

The model is trained on an NVIDIA Tesla V100 GPU with a minibatch of 2, where one image is from $\mathbb {D}_{S}$ (without tumor resection) and the other one is from $\mathbb {D}_{T}$ (with tumor resection). We use synchronized SGD and adopt Adam optimizer for optimization. The initial learning rate is 0.0002, and a *cosine* learning policy is employed. Weight decay of 0.0005 and momentum of 0.9 are used for training. The hyper-parameters $\lambda , \lambda _\text {rec}, \lambda _\text {advG}, \lambda _\text {advD}, \lambda _\text {segG}, \lambda _\text {segS}$ are empirically set as 1.0, 10, 1.0, 1.0, 1.0, 1.0.

For pre-processing, considering the varying resolutions of different data in the original MR images, all the MR images are re-sampled to $1\times 1\times 1$ $mm^3$. We then extract the patch of size [64, 96, 64] as network input. Random affine transformations (including random rotation, random scale, and random translation) are employed for data augmentation during training. We first train our improved 3D U-Net for 200 epochs with data from both $\mathbb {D}_{S}$ and $\mathbb {D}_{T}$. We then attach VAE-GAN and train for 200 epochs: We turn off the gradient update for the 3D U-Net in the first 100 epochs and let the model train the VAE-GAN only. We finished the training by turning the 3D U-Net gradient update on and letting the model train end-to-end. During the inference stage, we only apply the segmentation network to predict the hippocampus, and the generator could be discarded for computational efficiency.

### Evaluation metrics

In this study, several commonly used metrics including Dice Score Coefficient (DSC), Hausdorff Distance (HD), and Average Surface Distance (ASD) are adopted for evaluation of hippocampus segmentation with all compared approaches. Furthermore, We include precision, sensitivity, and specificity for quantitative comparison.

**Table 1:** DSC, HD(mm) and ASD(mm) of results by different comparative methods. T: the target domain dataset (BTR). S: the source domain dataset (NC). S(it): the source domain dataset with intensity transfer only. S$\rightarrow$T(it): synthetic images from the source domain (postoperative transfer and intensity transfer)

Model	Dataset	DSC	HD(mm)	ASD(mm)
3D U-Net	T	0.6971	17.20	1.82
3D U-Net	S+T	0.6986	10.90	3.32
SegNet	S+T	0.7199	10.66	1.39
Ours w/o VAE-GAN	S(it)+T	0.7354	5.06	0.83
Ours (full)	S$\rightarrow$T(it)+T	0.7429	9.40	1.13
Ours (full, train jointly)	S$\rightarrow$T(it)+T	**0.7546**	**4.68**	**0.75**

### Ablation study

In this section, we compare our model quantitatively through ablation studies on baselines as well as added components. 1) We train 3D U-Net as baseline segmentation model with $\mathbb {D}_{T}$ brain tumor only and one with both domains ($\mathbb {D}_{S}$ nasopharyngeal carcinoma and $\mathbb {D}_{T}$ with brain tumor). We evaluate models in each respect; 2) We use our improved segmentation network (SegNet) to train it with data from both domains. 3) We perform intensity transfer to align the intensity of $\mathbb {D}_{T}$ to $\mathbb {D}_{S}$ (IntAli) and train it on our SegNet. 4) We apply our VAE-GAN to synthesize postoperative images by transferring $\mathbb {D}_{T}$ images’ postoperative regions to images of $\mathbb {D}_{S}$, and we could thus use the generated images together with real images from $\mathbb {D}_{S}$ to train our segmentation model. At the same time, we also want to show that training the VAE-GAN and SegNet synchronously promotes each other even further. Therefore we train the networks (VAE-GAN and SegNet) separately such that when SegNet is trained, we freeze the gradient of the VAE-GAN. 5) This final experiment has the exact model configuration as 4) but this time we jointly train both VAE-GAN and SegNet such that mutual promotions take effect.

We report results on the dataset with brain tumors resection (BTR). The baseline experiments train with only $\mathbb {D}_{T}$ training set (55 images), and the rest of the experiments train on the combined training datasets (103 images).

The results are shown in Table 1. We can obtain the following observations: 1) Simply combining the images from the two domains (S + T) for a more extensive training set only brings negligible improvement of the DSC score compared with the target dataset $\mathbb {D}_{T}$ (from 0.6971 to 0.6986), and ASD score gets worse (from 1.82 to 3.32). This shows that naively combining multiple datasets from different domains is ineffective in handling the domain gap problem. 2) The modified 3D U-Net structure considerably helps with the overall performance (from 0.6986 to 0.7199), indicating that a more suitable backbone network could improve performance on a certain task. 3) Intensity alignment module could further improve the model by addressing the domain gap between $\mathbb {D}_{S}$ and $\mathbb {D}_{T}$. Performance on $\mathbb {D}_{T}$ can reach 0.7354, suggesting that intensity is a key factor in the segmentation performance. 4) When using our VAE-GAN for postoperative region transfer in addition to intensity alignment, the testing result on $\mathbb {D}_{T}$ can be further lifted to 0.7429 with some compensation from HD and ASD, suggesting postoperative region is another important information that helps with the segmentation performance. 5) Finally, we modified our networks’ training strategy from training them separately to training them jointly. We can see an even larger improvement in dice score: 0.7546 and produce the lowest HD and ASD scores. The increase in scores shows that training VAE-GAN with SegNet does induce a mutual promotion, which raises the segmentation accuracy as well as reduces its variance. All of the improvements check out our original hypothesis and demonstrate the effectiveness of our proposed methods.

**Table 2:** DSC, HD(mm) and ASD(mm) of results compared with baseline, MUNIT [22], DiscoGAN [23] and CycleGAN [21] using 2009-2019 data

	DSC	HD(mm)	ASD(mm)	Precision	Sensitivity	Specificity
3D U-Net Baseline	0.6971	17.20	1.82	0.74	0.63	0.99
MUNIT [22]	0.7053	14.34	3.12	0.69	0.77	0.99
DiscoGAN [23]	0.7310	9.32	1.98	0.68	0.75	0.99
CycleGAN [21]	0.7206	9.87	1.13	0.72	0.73	0.99
**Ours**	**0.7546**	**4.68**	**0.75**	0.72	0.77	0.99

**Table 3:** DSC, HD(mm), ASD(mm), precision, sensitivity and specificity of results compared with baseline, MUNIT [22], DiscoGAN [23] and CycleGAN [21] on our recent collected dataset from 2023

	DSC	HD(mm)	ASD(mm)	Precision	Sensitivity	Specificity
3D U-Net Baseline	0.6421	13.07	2.23	0.64	0.67	0.99
MUNIT [22]	0.6463	13.70	3.12	0.64	0.67	0.99
DiscoGAN [23]	0.6577	21.2	2.07	0.65	0.68	0.99
CycleGAN [21]	0.6744	18.01	2.08	0.69	0.67	0.99
**Ours**	**0.7215**	**6.72**	**1.06**	0.72	0.73	0.99

### Quantitative comparison

In this section, we compare our method with other state-of-the-art methods for image-to-image translation. MUNIT [22], DiscoGAN [23] and CycleGAN [21] are three popular GAN frameworks primarily used to learn transformations between images of different domains using unpaired datasets. Following our experimental setup, we first adopt these models to translate images from $\mathbb {D}_{S}$ to $\mathbb {D}_{T}$, and then use such synthetic images together with the real images from $\mathbb {D}_{T}$ to train the segmentation model. The results are shown in Table 2. By comparing the DSC score, we can see that our method outperforms MUNIT by 0.0493, DiscoGAN by 0.0236, and CycleGAN by 0.0340. We found out that MUNIT fails to learn different intensity levels between the two image domains and focuses on transferring the fine appearances. DiscoGAN, on the other hand, does perform well on intensity transfer. However, it fails to generate fine detail appearances. CycleGAN generates the best-looking images out of the three. However, just like MUNIT, it doesn’t do well in intensity transfer. Furthermore, all three GAN models fail to generate images with the postoperative region. Additionally, we included precision, sensitivity, and specificity for all comparison models. Results from sensitivity suggest that our method has the largest positive detection rate while results from specificity revealed that all models succeeded in predicting the background.

To showcase our model’s robustness to the test-of-time and different image modality, we included additional patient data from 2023 combined with the previously mentioned data. This new data set has a different imaging sequence of T1 C+ to pose a significant segmentation challenge. We followed the same experimental setup, and the results are shown in Table 3. Since we introduced a second type of light intensity into the dataset, the Dice score of all models dropped across the board as much as $\sim 0.08$ with a significantly worse HD value. Because our method contains the intensity transfer module, the model persisted in a promising result with the smallest drop in all metrics.

These comparisons demonstrate that explicitly synthesizing postoperative regions and intensity transfer is an effective scheme to overcome the domain gap problem for hippocampus segmentation after brain tumor resection.

### Qualitative comparison

**Fig. 5:**
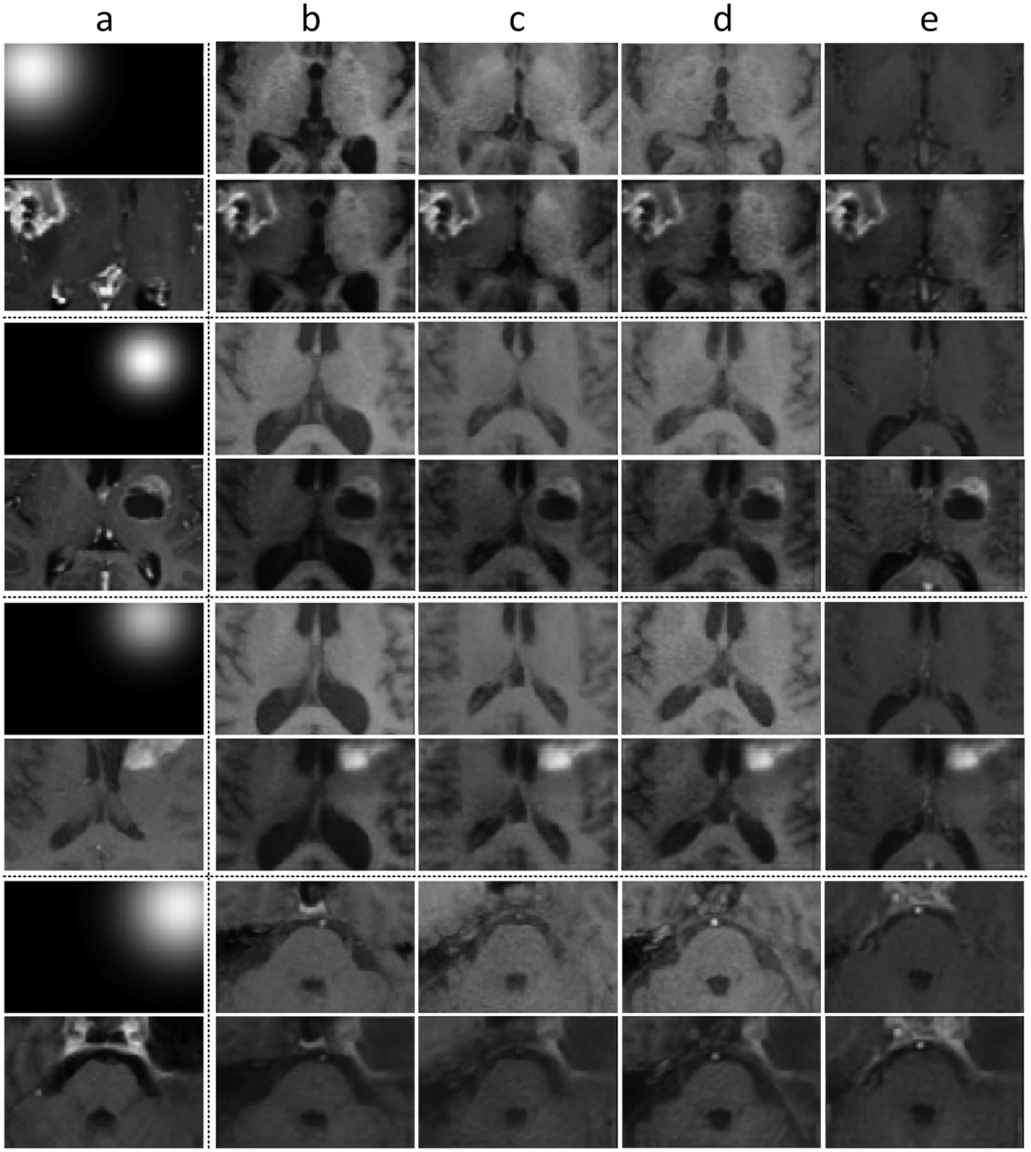
Visualization of postoperative image synthesis. (a) The images with postoperative regions and their weight map from $\mathbb {D}_{T}$. (b$\sim$e) Images from the source domain $\mathbb {D}_{S}$ and the synthesized images of our proposed method

**Postoperative region synthesis.** We show some visualization results of postoperative region synthesis in Fig. 5.

We randomly select images with the postoperative region from the target domain $\mathbb {D}_{T}$ as well as its corresponding Gaussian weight map as references. Then we transfer the postoperative region into the images from the source domain $\mathbb {D}_{S}$.

We can see that our proposed VAE-GAN can successfully transfer the postoperative region into an image without such a region. In addition, our method can automatically align the intensity between the synthetic image and the reference image, making the synthetic images more agreeable with the surrounding context. With these synthetic images as training samples, we can effectively improve the performance for hippocampus segmentation of patients after brain tumor resection.

**Fig. 6:**
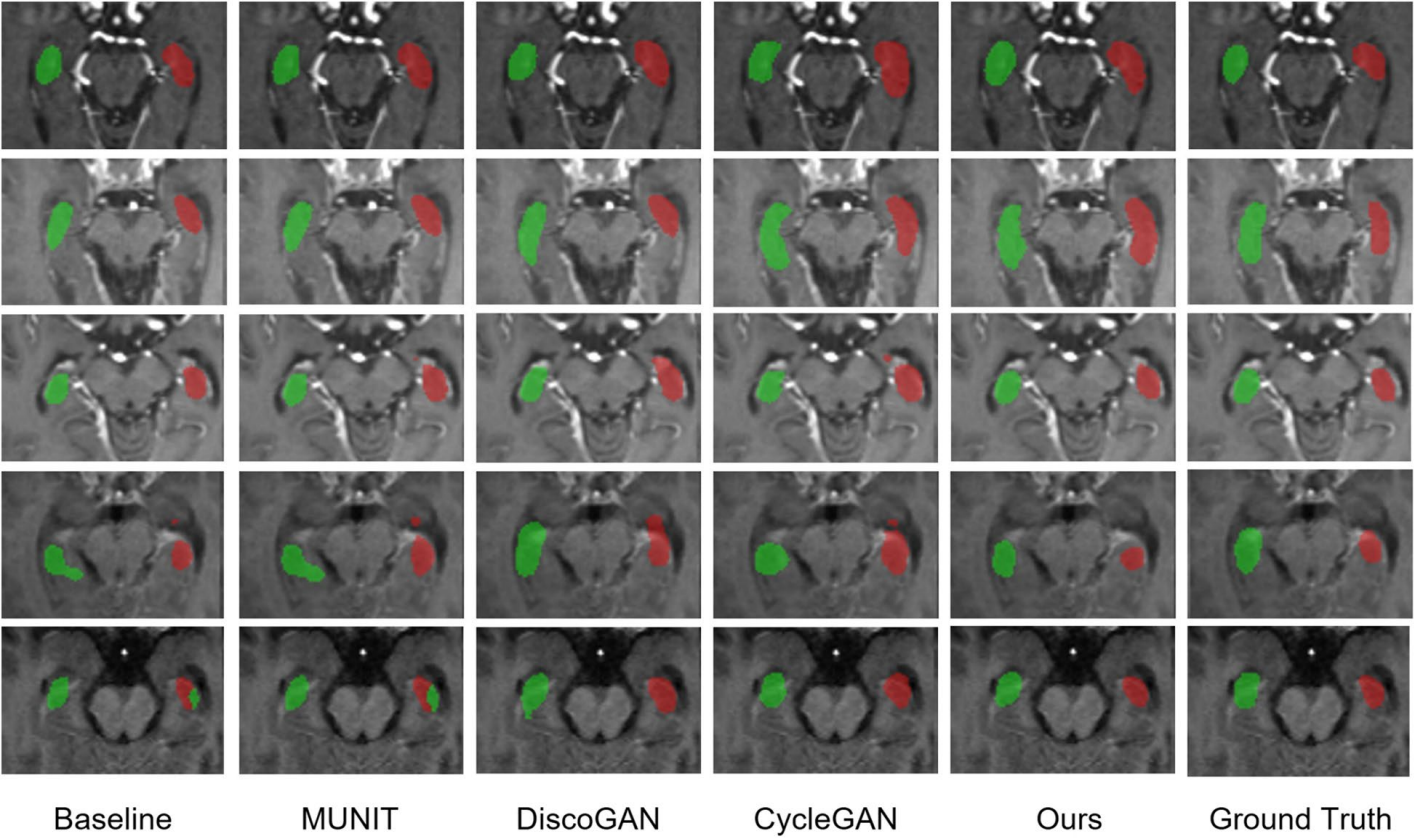
Visualization of each comparative method for hippocampus segmentation. Baseline: Only using images from $\mathbb {D}_{T}$ to train. MUNIT [22], DiscoGAN [23], CycleGAN [21]: Using synthetic images from the model and real images from $\mathbb {D}_{T}$ as training samples. Ours: Using synthetic images from our proposed VAE-GAN framework and real images from $\mathbb {D}_{T}$ as training samples

**Hippocampus segmentation.** We show some visualization results of some comparison methods for hippocampus segmentation on images from $\mathbb {D}_{T}$, including baseline, MUNIT [22], DiscoGAN [23], CycleGAN [21], and our proposed method. As illustrated in Fig. 6, in the first column, when training baseline model on $\mathbb {D}_{T}$, the segmentation result is significantly poorer compared to other methods on the right, which is consistent with the quantitative result. The quantitative result from MUNIT is closer to the baseline signifies that the domain gap problem, specifically intensity differences between two domains, is certainly an important factor to consider. There also exist distinct false positives (fourth row, first, second, and fourth col; fifth row, first, second col), and the cause of this is perhaps the insufficient training data and GAN not generalizing well on both domains. Our method, on the other hand, can generate the most superior results for hippocampus segmentation, outperforming the baseline and rest of the methods. This proves that our proposed VAE-GAN framework can synthesize realistic images with the postoperative region, and the synthetic images can benefit the hippocampus segmentation task.

## Conclusion and discussion

To generate more training data for accurately segmenting the hippocampus on the MR image of patients with tumor resection, we propose synthesizing the image with the postoperative region by image of other diseases (such as nasopharyngeal carcinoma). We design a novel end-to-end generation-segmentation framework, consisting of a VAE-GAN and a segmentation network. Given an image after tumor resection, our VAE-GAN could transfer the postoperative region to the image without tumor, and further align the intensity of two domains, making synthetic images more harmonious. Moreover, we design a modified 3D SEResUNet as the segmentation network. Extensive experiments demonstrate the effectiveness of the proposed framework in boosting the performance (+$\sim$5% DSC) of hippocampus segmentation on the MR image of patients after surgery.

There is still room to improve from this current work. In future projects, we will explore more factors that contribute to closing the “gap” performance across different image modalities. One possible way is using larger dataset sizes. We will also improve our VAR-GAN by incorporating better image modality adaptation methods. Additionally, it is possible to look into better segmentation solutions such as incorporating attention-based models. Finally, exploration will be made by looking into a one-for-all method to align and improve the postoperative transfer quality.

## Data Availability

The datasets (Nasopharyngeal carcinoma and Brain tumor) used during the current study are available from the corresponding author on reasonable request.

## Abbreviations

ASD: Average Surface Distance
ASPP: Atrous Spatial Pyramid Pooling
BraTS: Multimodal Brain Tumor Segmentation Challenge
BTR: Brain Tumors Resection
DSC: Dice Score Coefficient
GAN: Generative Adversarial Network
GPU: Graphics Processing Unit
HD: Hausdorff Distance
MR: Magnetic Resonance
MRI: Magnetic Resonance Imaging
MUNIT: Multimodal UNsupervised Image-to-image Translation
NC: Nasopharyngeal Carcinoma
ReLU: Rectified Linear Unit
SE: Squeeze Excitation
SGD: Stochastic Gradient Descent
VAE: Variational Encoder
